# Reducing complications of femoral neck fracture management: a retrospective study on the application of multidisciplinary team

**DOI:** 10.1186/s12891-023-06455-1

**Published:** 2023-04-29

**Authors:** Weiming Liang, Gang Qin, Lizhi Yu, Yingying Wang

**Affiliations:** The First Affiliated Hospital of Guangxi University of Science and Technology, Guangxi University of Science and Technology, 124 Yuejin Road, Liuzhou, 545001 Guangxi Province China

**Keywords:** Femoral neck fracture, Multidisciplinary team, Complication

## Abstract

**Background:**

Femoral neck fractures are associated with substantial morbidity and mortality for older adults. Multi-system medical diseases and complications can lead to long-term care needs, functional decline and death, so patients sustaining hip fractures usually have comorbid conditions that may benefit from application of multidisciplinary team(MDT).

**Methods:**

This is a retrospective cohort study that incorporates medical record review with an outcomes management database. 199 patients were included who had surgery for a new unilateral femoral neck fracture from January 2018 to December 2021 (96 patients in usual care (UC) model and 103 patients in MDT model. High-energy, pathological, old and periprosthetic femoral neck fracture were excluded. Age, gender, comorbidity status, time to surgery, and postoperative complication, length of stay, in-hospital mortality, 30-day readmission rate, 90-day mortality data were collected and analyzed.

**Results:**

Preoperative general data of sex, age, community dwelling and charlson comorbidity score of MDT group (n = 103) have no statistically significant difference with that of usual care (UC) group. Patients treated in the MDT model had significantly shorter times to surgery (38.5 vs. 73.4 h;P = 0.028) and lower lengths of stay (11.5 vs. 15.2 days;P = 0.031). There were no significant differences between two models in In-hospital mortality (1.0% vs. 2.1%; P = 0.273), 30-day readmission rate (7.8% vs. 11.5%; P = 0.352) and 90-day mortality (2.9% vs. 3.1%; P = 0.782). The MDT model had fewer complications overall (16.5% vs. 31.3%; P = 0.039), with significantly lower risks of delirium, postoperative infection, bleeding, cardiac complication, hypoxia, and thromboembolism.

**Conclusion:**

Application of MDT can provide standardized protocols and a total quality management approach, leading to fewer complications for elderly patients with femoral neck fracture.

**Trial registration:**

No.

## Introduction

Femoral neck fracture is a common injury in orthopedic practice which can cause significant morbidity and mortality [1]. The incidence of femoral neck fractures is increasing due to age-aging reasons, and the risk of fracture doubles every decade after age 50 [2]. Most hip fractures are associated with a fall, although other risk factors include osteoporosis, reduced level of activity, and chronic medication use [3, 4]. The 1-year mortality rate of femoral neck fracture can be up to 30% [5]. Half of the patients were unable to regain pre-fracture mobility, a fourth of whom require long term nursing home care before they had the ability to live independently [6].

Most femoral neck fractures occur in older adults who often have multi-system medical diseases and are at high risk of developing complications such as infection, delirium, and iatrogenic problems [7, 8]. These multi-system medical diseases and complications can lead to long-term care needs, functional decline and death.Surgical decision-making and perioperative management of elderly hip fractures require the joint participation of relevant multidisciplinary physicians including of not only orthopedic surgeons but also doctors of geriatrics, critical care medicine, anesthesiology, mental health department and rehabilitation medicine [9].

Agreed by the international Guidelines, the optimal treatment of hip fractures is immediate surgery for the reduction of the fracture and prosthetic replacement, enhancing the probability of better patient recovery [10]. Arthroplasty (Hemiarthroplasty and total hip replacement) is the treatment of choice for most older individuals who sustain a displaced femoral neck fracture [11]. Long waiting times before intervention will increase complications and mortality for patients with femoral neck fracture [12, 13]. Unfortunately, the surgery was delayed several days after the patient’s admission to hospital in many cases, which was seldom attributable to clinical reasons, but was more reasonably due to organizational challenges and bureaucracy [14]. The reasons for the delay could be as follows: patients with femoral neck fracture needed to spend a lot of time queuing for the preoperative examination, because the hospital did not open the preferential pathway for them;doctors of geriatrics waited until the next day to arrive for a consultation;the surgery was postponed due to the chief surgeon’s work schedule.

Multidisciplinary team(MDT) is a form of comanagement which has decreased inpatient complications and length of stay [15]. It refers doctors from more than 2 disciplines should conduct consultations for a certain disease, discuss the difficult problems in the diagnosis and treatment of the disease, and finally develop a reasonable and effective treatment plan.

In January 2020, we instituted the MDT program in which elderly patients with femoral neck fractures are admitted to a service comanaged by attending and resident physicians from the internal medicine and orthopaedic surgery departments. We hypothesized that MDT model focused on the care of elderly patients with femoral neck fractures will lead to fewer complications overall compared with usual care (UC).

## Methods

### Description of MDT and UC models

In UC model, the patient is treated by the orthopedic surgeon after admitted to hospital.The orthopedic surgeon asks the medical history in detail, conducts a systematic and comprehensive evaluation, and adopts general consultation. The relevant examination and treatment should be further implemented according to the consultation opinions. Surgery will be performed after the patient’s basic disease is stable and anesthesia consultation opinions is satisfactory.According to the patient’s condition after surgery, consultation from other departments will be performed if necessary.Patients with more medical diseases and more severe diseases will be sent to the intensive care unit after surgery. After surgery, the patient’s vital signs, mental state, feeding condition, blood routine, biochemical indicators(including of liver function, renal function, myocardial enzyme, electrolyte, blood gas analysis), induced flow rate and cardiopulmonary function were observed, and bilateral limb vascular ultrasound examination was performed. The affected limb was raised, and the quadriceps isolong contraction and ankle pump movement were guided.Prophylactic antibiotics were used within 24 h after surgery.Anticoagulation treatment with rivaroxaban is given routinely.Leaving bed was guided according to fracture type, surgical condition, and systemic condition. Patients with good wound healing, no hip pain, no serious complications, and no serious abnormalities in various laboratory indicators were admitted to discharge.

The MDT team was led by orthopedic surgeons, composed of attending doctors of geriatrics, critical care medicine, anesthesiology, mental health department and rehabilitation medicine. The MDT will evaluate the patient after admission, formulate a personalized examination and treatment plan, open the green channel, shorten the waiting time for examination, adjust the status of the patient to actively prepare for surgery, and shorten the time from admission to operation as far as possible. The surgical treatment plan was identical to the UC group. Patients with more medical diseases and more severe diseases will be sent to the intensive care unit after surgery.Rehabilitation medicine doctors guide the patient to exercise muscle strength and joint mobility, and guide the patient to get early out of bed. Isometric quadriceps contraction and ankle pump training began 6 h after surgery; knee flexion and straight leg elevation started 1 day after surgery; and walking training with the help of the walker started 2 days after surgery. Other postoperative diagnosis and treatment and discharge criteria were the same as the UC group.

The multidisciplinary team that evaluated the patients was composed of the same people for all patients. All surgeries were performed by the same surgeon.

### Study design

This is a retrospective cohort study that incorporates medical record review with an outcomes management database. Information for this database was collected on all patients with femoral neck fractures who complied with the inclusion criteria from January 2018 to December 2021.

### Ethical approval and consent

The study was conducted according to the Declaration of Helsinki and the International Conference on Harmonisation Tripartite Guideline on Good Clinical Practice. All patients provided written informed consent before participating. Approvals from Ethics Committee of the First Affiliated Hospital of Guangxi University of Science and Technology were obtained in December 2021(approval number:2021-LC076).

### Patients

Inclusion criteria: new unilateral femoral neck fracture patient aged more than or equal to 65 years. Exclusion criteria: fracture due to a high-energy trauma; pathological fractures;old fracture that occurs more than 6 weeks ago; periprosthetic femoral neck fracture. From the electronic database of our hospital, we identified 199 patients who had surgery for a femoral neck fracture from January 2018 to December 2021. Since the initiation of the MDT in January 2020, 96 patients from January 2018 to December 2019 were determined in UC model and 103 patients from January 2020 to December 2021 were determined in MDT model.

### Data collected

We collected demographic data, including name, date of birth and gender from the electronic database of our hospital. Inpatient charts (including admission notes, progress notes, operative dictations, consult notes, and discharge summaries) were reviewed to collect the date of admission, date and time of surgery, date of discharge, type of operative repair, comorbid diagnoses and complications. We used Charlson Comorbidity Index [16] to quantify patient comorbidity.

Complications included delirium, postoperative infection, renal insufficiency, bleeding, cardiac, hypoxia, thromboembolism, stroke. Postoperative infection included urinary tract infection, pneumonia, and surgical site infection. Bleeding included gastrointestinal, retroperitoneal, intracranial bleeding, hemorrhagic stroke, or wound hematoma. Cardiac included any new arrhythmia, acute myocardial infarction, or congestive heart failure.

### Statistical analysis

Differences in baseline variables and outcomes between the two models were compared via χ2 analysis for categorical variables, and the Fisher exact test was used for variables with expected cell values less than 5. Continuous variables were compared via the unpaired t test. Differences was considered significant at P < 0.05.

## Results

Characteristics of the two populations at Baseline are given in Table 1. In the two groups, the comparative differences in preoperative general data of sex, age, community dwelling and charlson comorbidity score were not statistically significant (P > 0.05).

**Table 1:** Characteristics of Patients at Baseline

Characteristic	MDT (n = 103)	UC (n = 96)	P value
Age, mean (SD), y	80.6(7.9)	81.2(8.3)	0.631
Male, %	41.2	39.6	0.683
Community dwelling, %	88.3	85.4	0.507
Charlson comorbidity score, mean (SD)	2.2(1.5)	1.9(1.7)	0.131

The data in Table 2 shows the differences with respect to outcomes between the two models of care. Patients treated in the MDT model had significantly shorter times to surgery (38.5 vs. 73.4 h;P = 0.028) and lower lengths of stay (11.5 vs. 15.2 days;P = 0.031). There were no significant differences between two models in In-hospital mortality (1.0% vs. 2.1%; P = 0.273), 30-day readmission rate (7.8% vs. 11.5%; P = 0.352) and 90-day mortality(2.9% vs. 3.1%; P = 0.782).The MDT model had fewer complications (16.5% vs. 31.3%; P = 0.039), with significantly lower risks of delirium, postoperative infection, bleeding, cardiac complication, hypoxia, and thromboembolism.

**Table 2:** Outcomes in the MDT and UC

Outcome	MDT (n = 103)	UC (n = 96)	P value
Time to surgery, mean (SD), h	38.5(18.6)	73.4(65.8)	0.028
Length of stay, mean (SD), d	11.5(5.6)	15.2(6.8)	0.031
In-hospital mortality, %	1.0	2.1	0.273
30-day readmission rate, %	7.8	11.5	0.352
90-day mortality, %	2.9	3.1	0.782
Complications overall, %	16.5	31.3	0.039
Delirium, %	11.7	23.9	0.032
Postoperative infection, %	7.8	13.5	0.047
Renal insufficiency, %	3.9	5.2	0.092
Bleeding, %	1.0	4.2	0.037
Cardiac, %	1.9	7.3	0.023
Hypoxia, %	3.9	9.4	0.046
Thromboembolism, %	1.0	6.3	0.031
Stroke, %	1.0	2.1	0.241

## Discussion

Our study shows that patients with femoral neck fracture treated in a MDT model of care experience better outcomes than those in the UC model. Specifically, patients in the MDT model underwent surgery approximately one and a half days earlier than those in the UC model. Patients in the MDT model were admitted to discharge approximately four days earlier than those in the UC model.Our study shows substantial promise in decreasing inpatient complications, with MDT patients experiencing a 16.5% complication rate overall vs. 31.3% for UC patients. The complications that were significantly lower in the MDT model were delirium, infection, bleeding,cardiac complications, hypoxia, and thromboembolism. There were no significant differences between two models in In-hospital mortality, 30-day readmission rate and 90-day mortality.

Surgical treatment can avoid the occurrence of bed-related complications in femoral neck fracture patients and reduce the mortality rate [17]. If the patient conditions permit, surgical treatment within 48 h after the injury has become a consensus [11]. Delayed surgery can significantly increase the incidence of complication [12, 13]. However, repeated preoperative consultation and examination of patients will delay the time, and some patients will have various complications while waiting for surgery, losing the timing of surgery [18]. For example, the preoperative chest CT examination indicated that the patient had pneumonia, and then the respiratory department consultation was conducted; after a few days of treatment for pneumonia, the chest CT was reviewed, and then the respiratory department consultation was conducted again; moreover, each consultation or examination might be delayed for one day. Such patients are often complicated with serious medical diseases, and timely and reasonable preoperative evaluation and perioperative management determine the success or failure of the treatment, so it is imperative to implement individualized treatment involving multiple disciplines. The American Society of Anesthesiologists (ASA) classification is strongly associated with medical problems in the perioperative period following hip fracture surgery in the elderly [14]. Patients identified as being at higher risk (in ASA class 3 or 4) preoperatively should be closely managed medically so that perioperative medical complications can be managed and evolving medical issues can be addressed in a timely fashion.

The concept of multidisciplinary team(MDT) was first proposed by the M.D.Anderson Cancer Center in the United States. It refers to discussing the difficult problems in the diagnosis and treatment of patients through consultation in more than two disciplines, and finally formulating a reasonable and effective treatment plan [19]. The concept makes the traditional individual empirical diagnosis and treatment mode into a careful, accurate and reasonable standardized diagnosis and treatment team mode, maximize to avoid the disadvantages of the too fine modern medical branch.It is efficient and convenient, avoid the patients repeated consultation and examination, allow patients enjoy one-stop medical services, and is also an important content of the implementation of postoperative rehabilitation concept.

The purpose of exploring MDT in the treatment of elderly femoral neck fractures is to integrate relevant medical resources, optimize processes, and rationally select treatment plans, so that patients can be treated safely with surgery as soon as possible, and reduce the complication rate and mortality. Recent studies have suggested the management model of MDT results in shorten time to surgery, shorter length of stay, lower complication rates and lower readmission rates [20–22], whose results are similar to ours.The composition of the MDT was similar in previous literature. Loizzo M et al. reported that the collaboration between healthcare system management, orthopedic specialists, geriatric specialist and physical therapists was needed to drive shorter days of hospitalization and better overall patient health outcome by performing surgery as soon as possible [22]. Lau TW et al. reported that their team was composed of not only surgeons, physicians, anaesthetists and nurses, but also the rehabilitation doctors, nurses, therapists and medical social workers in a rehabilitation hospital [9]. In our MDT model, orthopedic surgeons was the leader, and geriatric specialist was responsible for the management of multi-system medical diseases and complications.Anesthesiologist assessed the anesthesia risk and administered anesthesia. Doctors of critical care medicine and mental health department would participate according to the patient’s condition.

Advanced age and surgical trauma are well-recognized risk factors for post-operative delirium. Jin et al. retrospectively studied 258 patients with femoral neck fracture data, and found the incidence of postoperative delusion was 17.4% [23]; The mortality rate of patients who develop postoperative delusion is three times as much as the patient without postoperative delusion [24, 25]. In our study, The incidence of postoperative delusion in the MDT group was only 11.7%, which was significantly lower than the 23.9% of patients in the UC group.

Elderly patients with femoral neck fracture have not fully recovered at discharge, and mostly are clinical healing. The functional recovery of patients largely depends on good rehabilitation treatment.In the past, insufficient attention was paid to the postoperative rehabilitation of such patients, which can lead to artificial joint dislocation, malunion, joint stiffness, deep vein thrombosis and other complications [26]. The MDT model included the rehabilitation physician to provide rehabilitation treatment and guidance to such patients, so that the patients could get out of bed and exercise as soon as possible, and restore the affected limb function to the maximum extent.

In the MDT diagnosis and treatment, the most beneficial group is the high-risk patients with poor systemic condition. Under the cooperation of multidisciplinary doctors, we could implement individualized management in the perioperative period, actively conduct preoperative evaluation, timely and effectively intervene for the combined medical diseases, adjust the functions of patients to the best state for surgical treatment, and finally obtain a good prognosis [20]. Treatment of high-risk femoral neck fracture patients will be difficult to rely on a single discipline. Only by breaking the boundaries of the specialty, with the strength of multidisciplinary comprehensive treatment, and having a comprehensive understanding of the disease, can we provide patients the best treatment plan and improve the doctor-patient relationship, which will also be an inevitable trend of the development of large general hospitals.

There was no significant differences between two models in In-hospital mortality, 30-day readmission rate and 90-day mortality. In this regard, we believe that although patients can benefit from MDT, the extent of the benefit is not yet large enough to affect mortality and 30-day readmission rate.Similar studies also showed that significant differences in mortality cannot be caused by MDT model [15, 26, 27]. It means that MDT models of care may improve short-term outcomes for patients with femoral neck fracture, but it may not yield longer-term benefits. Perhaps long-term prognosis is closely related to the long-term control of multi-system diseases, which is not easily affected by MDT models. More clinical data are needed to confirm this opinion.

Besides, according to our experience, there are many problems that need to be overcome in the specific implementation process. The cognitive differences between disciplines might bring about controversy over treatment options. The cooperation could not operate for a long time due to the lack of performance incentive mechanism. The implementation of MDT needs the support of the hospital management, and a reasonable full top-level design is indispensable.

This study has several limitations. First, our study is a retrospective cohort study which depends on data available from medical record review for identification of comorbidity and complications. These limitations might affect both models and had influence in comparing outcomes between the two groups.Second, different outcomes between two groups may be attributable to some thing other than the model of care, such as surgical protocols, surgical approach, unmeasured patient characteristics, or nursing care, which were not measured in this study. Our study failed to analyze this information even further. Thir, our medical record review was unblinded, which could have led to bias in determining complications.

In conclusion, care of MDT model can provide standardized protocols and a total quality management approach, leading to fewer complications for elderly patients with femoral neck fracture. Replication of MDT model may improve outcomes for those patients with a common and serious condition. More multi-center, prospective, randomized controlled clinical trials are needed to confirm our results and to improve the management process of MDT.

## Data Availability

The data that support the findings of this study are available from the corresponding author upon reasonable request.

## Abbreviations

MDT: Multidisciplinary team
UC: Usual care
